# Deeper Insight into Photopolymerization: The Synergy of Time-Resolved Nonuniform Sampling and Diffusion NMR

**DOI:** 10.1021/jacs.2c05944

**Published:** 2022-07-19

**Authors:** Kristina Kristinaityte, Adam Mames, Mariusz Pietrzak, Franz F. Westermair, Wagner Silva, Ruth M. Gschwind, Tomasz Ratajczyk, Mateusz Urbańczyk

**Affiliations:** † Institute of Physical Chemistry, Polish Academy of Sciences, Kasprzaka 44/52, 01-224 Warsaw, Poland; ‡ Faculty of Chemistry and Pharmacy, Univeristy of Regensburg, Universitätsstraßze 31, 93053 Regensburg, Germany

## Abstract

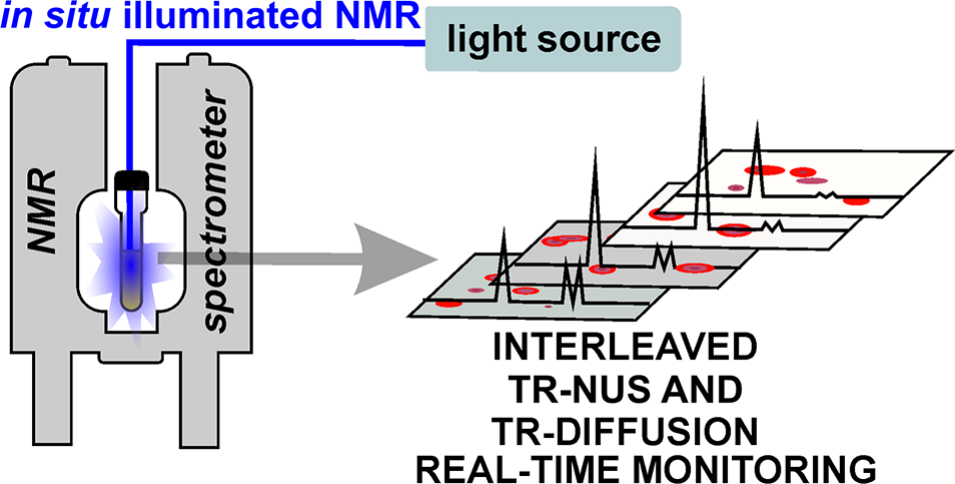

The comprehensive real-time in situ monitoring of chemical processes is a crucial requirement for the in-depth understanding of these processes. This monitoring facilitates an efficient design of chemicals and materials with the precise properties that are desired. This work presents the simultaneous utilization and synergy of two novel time-resolved NMR methods, i.e., time-resolved diffusion NMR and time-resolved nonuniform sampling. The first method allows the average diffusion coefficient of the products to be followed, while the second method enables the particular products to be monitored. Additionally, the average mass of the system is calculated with excellent resolution using both techniques. Employing both methods at the same time and comparing their results leads to the unequivocal validation of the assignment in the second method. Importantly, such validation is possible only via the simultaneous combination of both approaches. While the presented methodology was utilized for photopolymerization, it can also be employed for any other polymerization process, complexation, or, in general, chemical reactions in which the evolution of mass in time is of importance.

## Introduction

The design of novel functional materials requires a comprehensive insight into the chemical reactions or processes in which these materials are synthesized, fabricated, or functionalized. The above statement is particularly valid in the case of polymerization processes, as tuning the polymerization toward the development of products would be significantly facilitated by the knowledge of what is happening during the reaction.^1−3^ One of the most important variants of polymerization is photopolymerization, which is even more challenging for the investigator, as the illumination conditions are often difficult to control. This often causes problems with the reproducibility of the data on different setups.^4−7^ Thus, the knowledge of the fate of the photoreaction as photopolymerization progresses would be of particular value.

One of the most comprehensive analytical methods is NMR spectroscopy, which can deliver in-depth insight into molecular structures.

In recent years, we have witnessed a rapid growth of new NMR-based reaction monitoring methods.^8−16^ In particular, two main concepts have recently made significant progress: ultrafast (UF)^17,18^ and time-resolved (TR) methods.

Both methods arose concomitantly, and they possess somewhat orthogonal strengths and weaknesses.^19^

While the UF method allows much faster sampling leading to much better time resolution, the TR methods are more stable in out-of-equilibrium conditions. Therefore, the TR is a more optimal choice for photopolymerization monitoring, where the light source might cause additional disturbances in the system.

Time-resolved NMR was first introduced by Mayzel et al.^20^ for multidimensional nonuniform sampled NMR (TR-NUS). The concept of this method is based on a long nonuniform sampling schedule that covers the entire reaction time. Afterward, the data are separated into overlapping subsets from which spectra are reconstructed. As mentioned earlier, the overlapping subsets guarantee a high temporal resolution, while a single spectrum is time-averaged according to the size of the subset from which it was reconstructed. This method has been utilized widely to study many interesting systems.^21−25^ Interestingly, the method can be combined with an interleaved (IL) acquisition, where many different TR-NUS experiments are acquired in a parallel mode by switching between pulse sequences after each registered NUS point.^26^

A very useful progeny of TR-NUS is time-resolved diffusion ordered spectroscopy (TR-DOSY).^27,28^ This method combines the concept of the acquisition of TR-NUS data with the permutated DOSY (p-DOSY) approach^29^ by substituting the random sampling of the indirect dimension with randomly generated pulsed-field gradient strength in pulsed gradient stimulated echo (PGSTE) experiments. This method has recently been used by Fillbrook et al.^30^ to study the photoinduced electron/energy transfer reversible addition–fragmentation chain transfer (PET-RAFT) polymerization of methyl acrylate. This approach allowed the fate of the reaction and the evolution of the average molecular mass of the reactants to be followed. While the TR-NUS is related to the structure, the TR-DOSY offers information on how this structure behaves in time. A combination of analytical methods can often provide a correlation between parameters delivered by these methods and information that cannot be obtained when employed separately.

Interestingly, the TR-NUS and TR-DOSY methods have never been used together to study a particular system. Here, we combined these methods into one tool. We demonstrated that this combination is an excellent methodology for the comprehensive monitoring of photopolymerization.

To avoid utilizing an overly simplified model as a system for monitoring, a photopolymerization of bis-anthracene-based systems was chosen. The system is very interesting, as photopolymerization of aromatic sheets, including anthracenes, is being employed as building blocks for the design of various photofunctional materials.^31^ Therefore, an in-depth understanding of the photopolymerization of this type of system is crucial.

In this work, we demonstrate the feasibility and advantages of the interleaved acquisition of both methods to study the photopolymerization of bis-anthracene derivatives, specifically *N*,*N*-bis(anthracen-9-ylmethyl)butane-1,4-diamine (H2banthbn).^32−36^

## Experimental Section

### UV Illumination Setup

UV illumination was performed by a 365 nm (9 nm fwhm) fiber-coupled LED (M365FP1) purchased from Thorlabs. The maximum power of 15.5 mW at the end of the optical fiber was measured at a 1400 mA maximum current, using 1 m of FT400EMT Ø400 μm core, 0.39 NA multimode fiber. During all experiments, 3 m length FT1000UMT (Thorlabs) Ø1000 μm core, 0.39 NA multimode fiber was used. The optical fiber tip was polished using sandpaper to illuminate the sample more homogeneously. The LED current was set to 500 mA using a T-cube LED driver (LEDD1B), also bought from Thorlabs.

The optical fiber was fixed to the coaxial insert and placed in the Wilmad screw-cap NMR tube through the septum to avoid contact with oxygen after deoxygenating the sample. A 420 μL amount of the solution was chosen as optimal for the full illumination of the sample.

### Sample Synthesis and Preparation

The synthesis of H2banthbn was carried out according to the literature protocols with some modifications.^37,38^ Antraldehyde (1 g, 4.85 mmol) was dissolved in fresh distilled tetrahydrofuran (THF)/methanol (v/v = 30 mL/20 mL) mixed solvents under an inert atmosphere. Butane-1,4-diamine (213 mg, 2.42 mmol) in 10 mL of methanol was added dropwise. The mixture was stirred for 24 h at room temperature. The resulting precipitate was filtered and washed with cold methanol to remove excess antraldehyde. The crude imine was dissolved in fresh distilled dichloromethane (DCM)/methanol (v/v = 60 mL/20 mL) mixed solvents, and sodium borohydride (189 mg, 5 mmol) was added. After stirring at room temperature for 12 h, the yellow crystals were filtered. The product was purified by recrystallization in THF/pentane. Obtained: 998 mg, yield = 88% of yellow crystals. ^1^H NMR (400 MHz, CDCl_3_): δ ppm 8.39 (s, ^1^H), 8.35–8.29 (m, 2H), 8.02–7.98 (m, 2H), 7.48 (ddd, *J* = 15.5, 8.5, 1.3 Hz, 4H), 4.71 (s, 2H), 2.90 (s, 2H), 1.66 (s, 2H). ^13^C NMR (101 MHz, CDCl_3_): δ ppm 131.9, 131.5, 130.2, 129.1, 127.1, 126.0, 124.9, 124.1, 50.4, 45.8, 28.0. HRMS: *m*/*z* calcd for C_34_H_32_N_2_ [M + H]^+^ 469.2644, obtained 469.2643.

For the measurements, the 3 mg (6.4 × 10^–3^ mmol, *Cm* = 4.8 mM) of the H2banthbn was dissolved in 0.75 mL of deuterated dichloromethane (99.6% D, ampules purchased from Deutero GmbH). Then, 420 μL of the solution was poured into a Wilmad screw-cap NMR tube. Subsequently, the sample was degassed through three freeze–pump–thaw cycles to remove oxygen.

### NMR

All NMR experiments were performed on a Bruker AVANCE II 300 MHz spectrometer equipped with a BBI 300 MHz W1 5 mm z-gradient probe with a BVT-3000 temperature controller. The spectrometer was controlled via the TOPSPIN 3.2 program.

The acquisition protocol was based on the TReNDS^26^ acquisition script modified by substituting the standard ^1^H experiment with the stebpgp1s1d pulse sequence. The illumination was turned on during the entire acquisition. All experiments were performed with the temperature controller set to 298 K.

#### Time-Resolved Diffusion NMR

The stebpgp1s1d pulse sequence was used as a base for the acquisition of the TR-DOSY experiment. The acquisition parameters for interleaved acquisition were as follows: Δ: 50 ms, δ: 3 ms, pulse length: 9.5 μs, relaxation delay: 2.5 s, number of scans: 8. For the noninterleaved data sets, the parameters were slightly different: Δ: 50 ms, δ: 3 ms, pulse length: 9.5 μs, relaxation delay: 3 s, number of scans: 4.

The pulsed-field gradient strength for all data sets was set as a shuffled logarithmically spaced array of 32 values from 5.63 to 35.47 G/cm. The range was optimized for the diffusion coefficient in the system. The resulting gradient array was repeated 17 times to cover the reaction’s progress. This will guarantee that every frame used in the data processing will contain the same set of gradients.

#### Time-Resolved Nonuniform Sampling

The TR-NUS acquisition was based on the hsqcgpph pulse sequence. The acquisition parameters were as follows: relaxation delay: 2.5 s, number of scans: 32, indirect dimension spectral width: 8 ppm, centered at 127 ppm, maximum evolution points in indirect dimension: 64. The sampling schedule was generated using a sampling generator in the MDDNMR package^39^ with shuffle mode and the relaxation weighting parameter T2 set to 0.07. The effect of the choice of the nonuniform sampling scheme type (e.g., Poisson-gap or random) in the case of spectra of high sparsity (as in this work) should be negligible.^40^

### Data Analysis

The TR-NUS spectra were reconstructed by the TReNDS program using the IRLS algorithm^41^ with virtual echo.^42^ The frame size was set to 54 points with a 52-point overlap. We have applied exponential weighting for direct (5 Hz) and indirect (1 Hz) dimensions. To save time and memory, the spectrum was trimmed in the direct dimension to the region 9–6 ppm.

Further, the analysis was performed in the Python 3.8 environment using the following packages: nmrglue,^43^ jupyter-lab,^44^ NumPy,^45^ SciPy,^46^ and Matplotlib.^47^

The diffusion data were imported using nmrglue. Each spectrum was processed the same way: zero-filled to 131 072 points, weighted exponentially by 2 Hz, and Fourier transformed. After that, the region 9–6 ppm (see the yellow rectangle in Figure 2(a)) was integrated and stored. The integrals were divided into subsets of 32 points that overlapped by 31 points. Each subset was used to fit the diffusion coefficient using the monoexponential model:
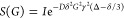
1where *S* is the integral of the peak intensity, *G* is the pulse field gradient strength, δ is the time of the *G*, Δ is diffusion mixing time, and γ is the gyromagnetic constant of ^1^H.

**Figure 1:**
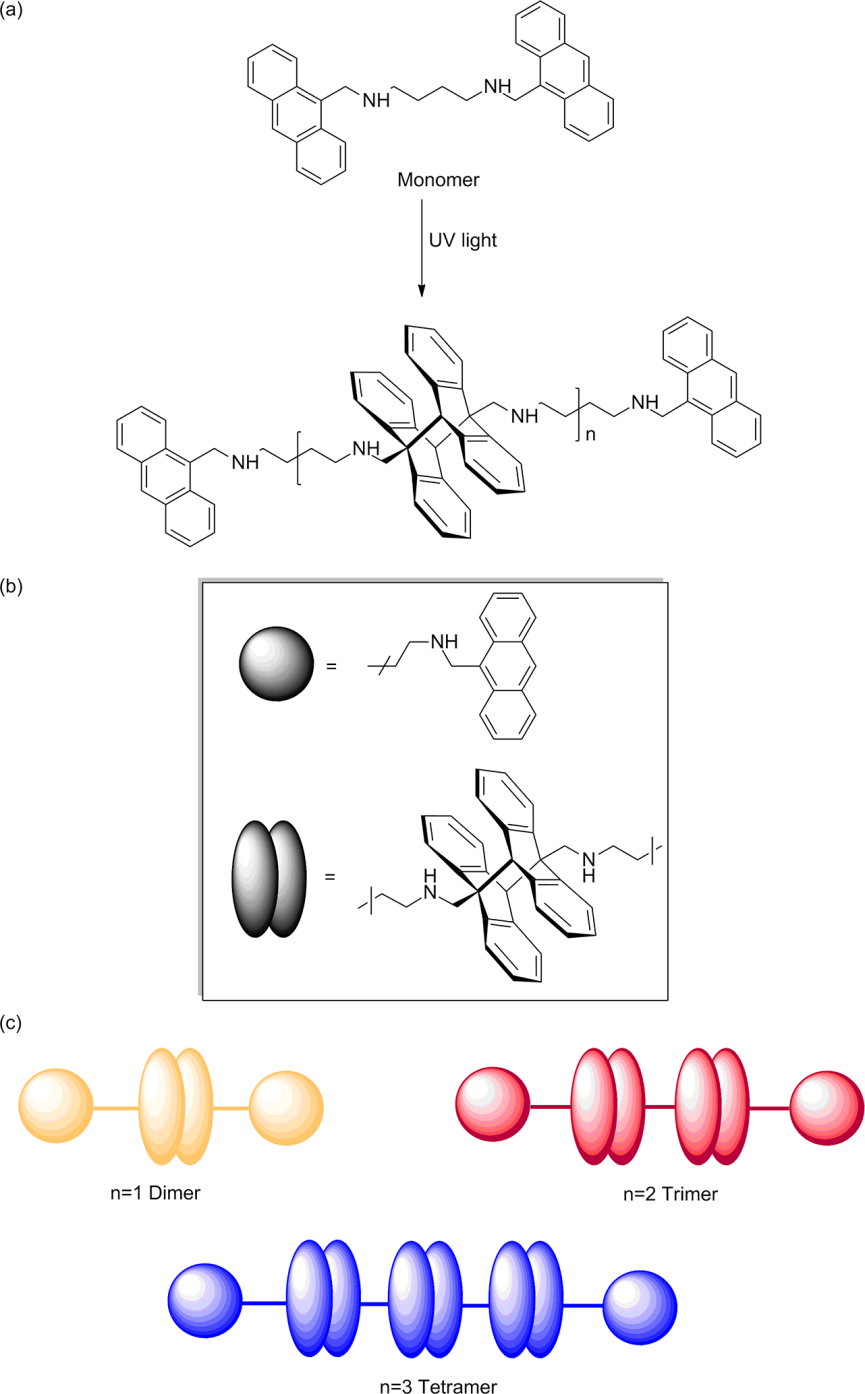
(a) General scheme of photopolymerization of H2banthbn. (b) Schematic representation of building blocks of *n*-mers. (c) Observed soluble products of the reaction.

**Figure 2:**
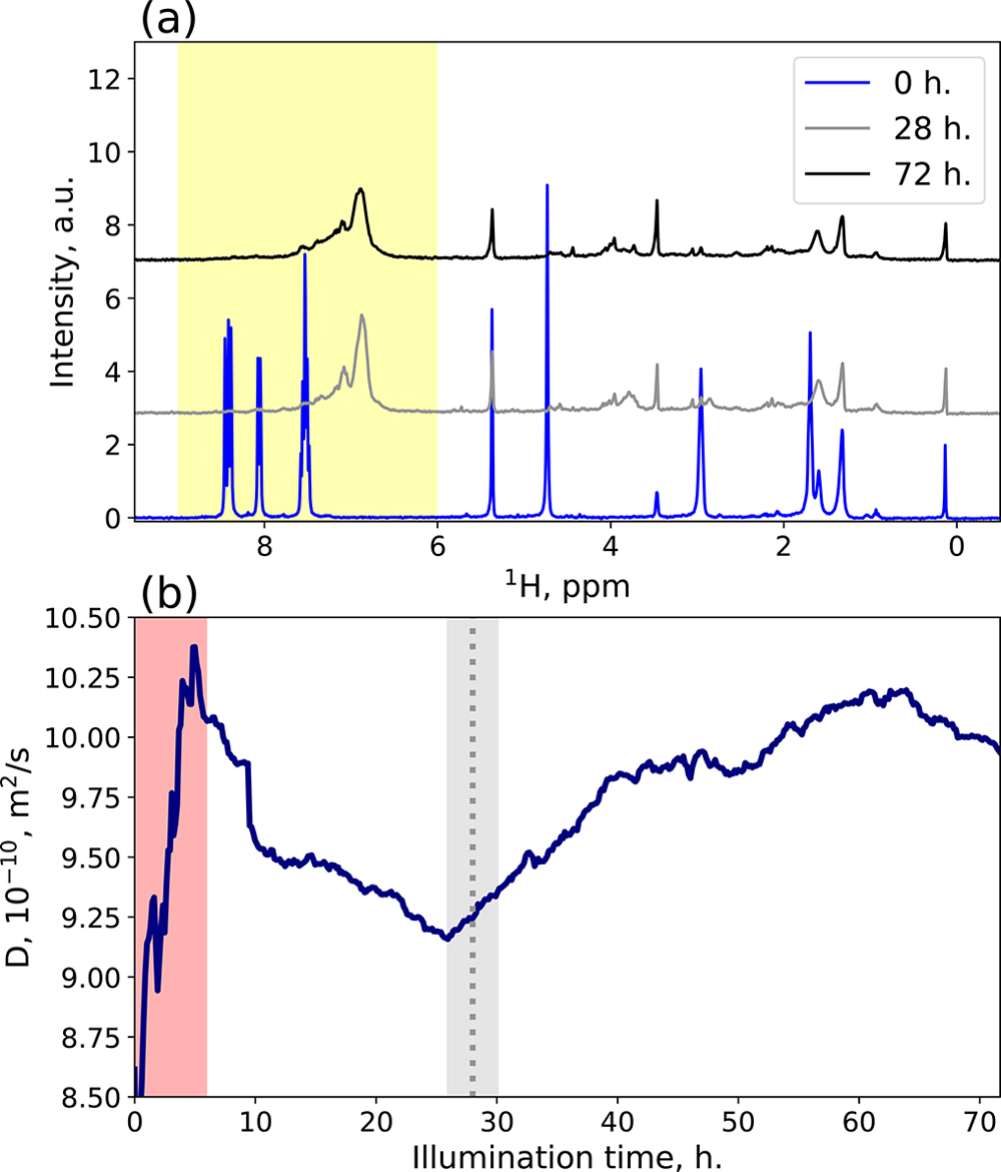
(a) ^1^H spectra taken from the PGSTE experiment at the gradient strength equal to 7.32 G/cm. The blue line represents the start of the reaction, the gray line the spectrum closest to the moment when the average mass was the highest, and the black line the end of the reaction. The area highlighted in yellow shows the analysis region used for the diffusion fitting. (b) Average diffusion coefficient calculated from the highlighted area in (a). The red highlighted square shows the disturbance in the diffusion coefficient caused by heating of the sample from the switched-on light source. The dotted vertical line indicates the time of the middle spectrum from the (a) pane, while the gray area represents the time-averaging of a single frame.

In theory, by applying the inverse Laplace transform (ILT),^48,49^ we should be able to track individual reactant evolution in time. Unfortunately, the ILT could not distinguish separate peaks. Therefore, we have decided to use single-exponential fitting only (shown in Figure 2(b)).

**Figure 3:**
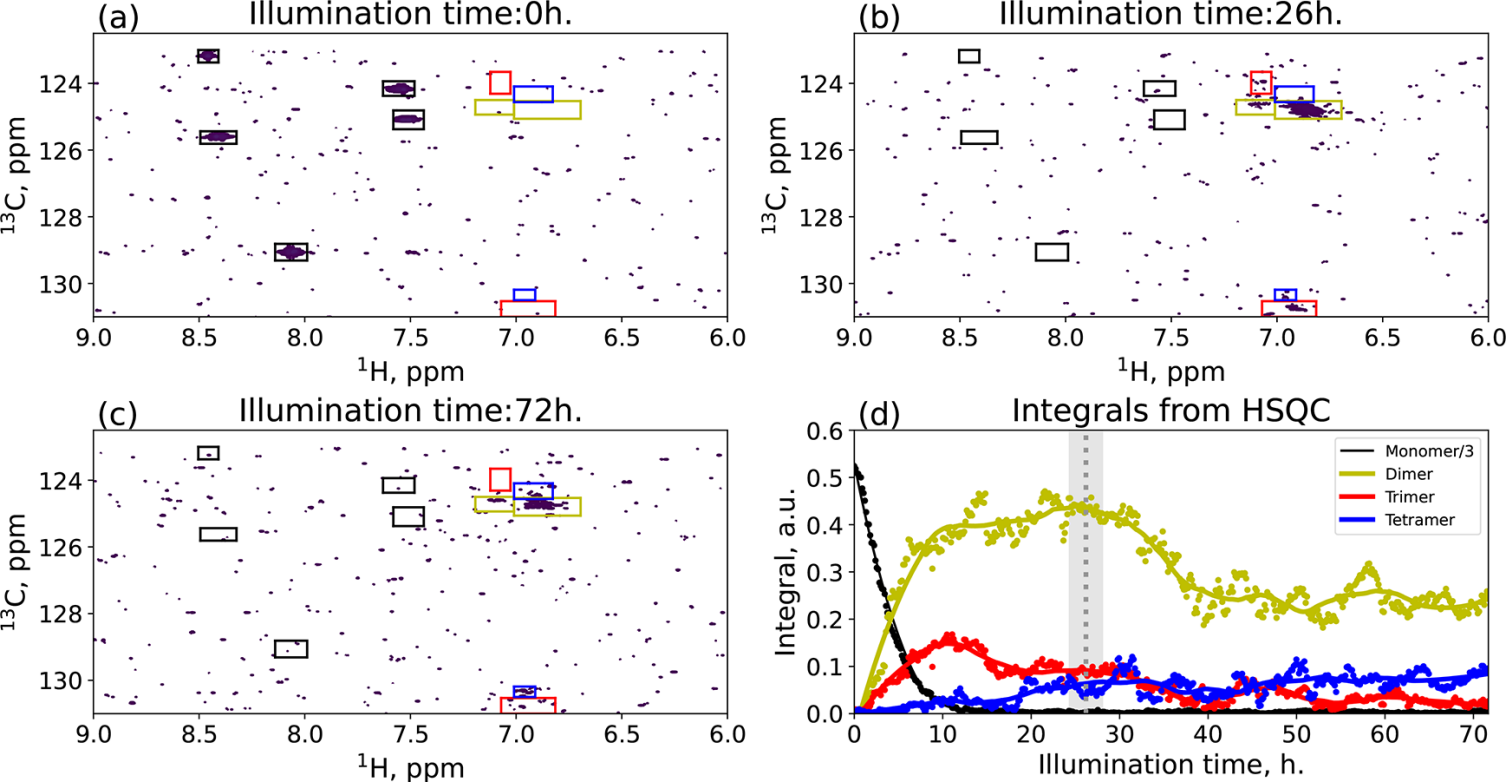
(a–c) HSQC spectra of the reacting mixture at three different time windows: (a) before illumination, (b) time at the maximum average weight, and (c) at the end of the reaction. The colored boxes represent the area where we have integrated the spectra. The color and label show the specific *n*-mer. The integrals from the boxes are shown in (d). The dots show the “raw” integrals, while the continuous lines are the data after the Savitzky–Golay filter. The dotted vertical line shows the time of the (b) pane, while the gray area represents the time-averaging of a single frame. To make the figure easier to read, the integral of the monomer is divided by a factor of 3.

After reconstruction, TR-NUS data were imported using nmrglue, and 11 regions were selected for integration (see boxes in Figure 3(a)–(c)). The regions were gathered into four groups, according to the same time-dependence profile. The groups were then assigned to the possible *n*-mers, and the signals’ intensity from each group was summed up. The resulting time dependence of each reactant’s peak intensity is shown in Figure 3(d). The next step was to recalculate the average mass of the system. To do this, each time-dependent profile of reactant concentration was smoothed by applying the Savitzky–Golay filter,^50^ and then, for each frame, the intensities were normalized by the integral of the whole region. With this approach, we calculated the average mass of the system as a function of time (⟨*M*⟩_HSQC_(*t*)) using a simple equation:
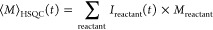
2where, *I*_reactant_(*t*) is the normalized integral of each reactant, while *M*_*reactant*_ is its mass.

**Figure 4:**
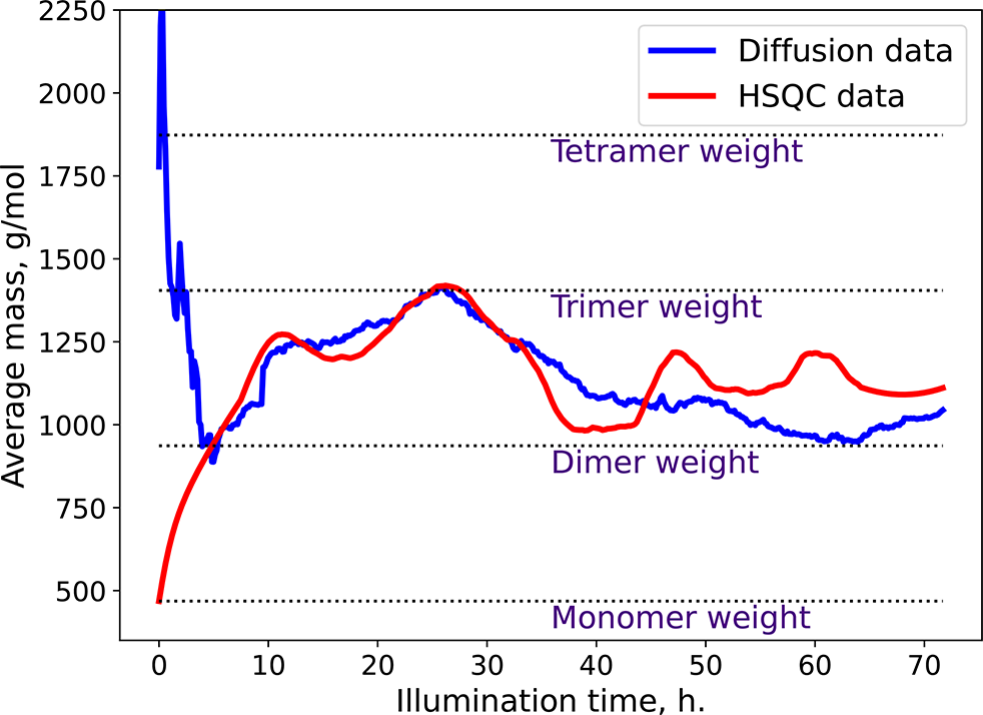
Time dependence of the average mass calculated by TR-DOSY (blue) and TR-NUS (red) methods.

The diffusion coefficients (*D*) can be transformed into molecular mass (⟨*M*⟩_D_ by following this equation:^51^
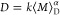
3where *k* and α are empirical scaling coefficients. We calculated those coefficients by substituting ⟨*M*⟩_D_ in eq 3 with ⟨*M*⟩_HSQC_ and fitting the equation for *t* > 6 h (to avoid the disturbances from the switched-on light source). The resulting comparison between average molecular mass values calculated from both methods is presented in Figure 4. Additionally, the two additional TR-DOSY experiments were analyzed, to confirm the correctness of the approach. The first one was following the same reaction as the interleaved one, and the second one was monitoring the dimerization of anthracene in identical conditions. From those experiments, we determined diffusion coefficients of anthracene, dimer-anthracene, and the dimer of H2banthbn. With three diffusion coefficients of three molecules of known masses, we have calculated the parameters *k* and α from eq 3 and established the average mass of H2banthbn photopolymerization (as shown in Figure SI.1).

## Results and Discussion

We have studied the photopolymerization of H2banthbn. The scheme of the reaction is shown in Figure 1. Photodimerization of amine-based anthracene derivatives is well known in the literature, and head/tail isomers of dimers are the preferred products.^52−54^

We started our analysis with an approach similar to the one suggested by Fillbrook et al.^30^ We have monitored the reaction using the TR-DOSY method. After the processing, we obtained our system’s average diffusion coefficient (see Figure SI.1).

For the reaction analysis, it is more convenient to use average mass than diffusion coefficients. We addressed this issue with different approaches for interleaved and sequential acquisitions. We have used the masses and diffusion coefficients of anthracene, the anthracene dimer, and H2banthbn for the calibration curve (the result is shown in Figure SI.1). The resulting time dependence of the average mass revealed interesting behavior. It steadily rose for the first 28 h until it achieved the highest average mass value: 1476 g/mol. Then the mass started to decrease slowly. Such behavior suggests that in the first 28 h of the reaction there is a mixture of products ranging from dimer to tetramer (the highest average mass is slightly larger than the mass of the trimer). After that, the higher mass polymers formed that precipitated from the solution (reducing the average mass of the reaction mixture). However, as the inverse Laplace transform analysis cannot distinguish separate forms in this data set, one cannot monitor the particular products.

We have combined TR-NUS HSQC and TR-DOSY into a single interleaved acquisition to overcome this limitation. The results from interleaved TR-DOSY were in significant agreement with the previous measurement. This time the TR-NUS HSQC experiment supported the data interpretation.

In the TR-NUS HSQC, we have analyzed all the peaks in the region 9.0–6.0 ppm (see Figure 3). We found out that the time dependency of those peaks forms four distinct groups. Each group is highlighted with a different color in Figure 3. The first group’s intensity was constantly decaying during the reaction. These regions were assigned as a monomer (black; ^13^C/^1^H: 123.00/8.46 125.60/8.41 129.00/8.05 124.17/7.55 125.07/7.52 ppm). The other regions were trickier to assign. The peaks’ intensity in the yellow regions (^13^C/^1^H: 124.75/6.86, 124.30/6.91 ppm) is rising the fastest at the beginning of the reaction, suggesting that they come from a dimer. The integral of the red regions (^13^C/^1^H: 124.00/7.06, 130.78/6.94 ppm) has a slower rise, and its maximum is at the moment when the monomer is depleted. We assume that this group corresponds to the trimer, as it needs both the dimer and the monomer in the reaction mixture to be formed. The peaks from the last groups (blue; ^13^C/^1^H: 124.70/7.09, 130.29/6.95 ppm) are characterized by steady growth throughout the reaction, suggesting that they can be assigned to a tetramer. Additionally, we can see a decline in the intensity of dimer and trimer signals. The first one is associated with the formation of the tetramer, while the second one forms insoluble higher *n*-mers. The precipitation was confirmed by the picture of the NMR tube after the reaction (see Figure SI.2) and the intensity loss of ^1^H spectra (see Figure SI.3).

The diligent reader might notice that the peaks shown in Figure 3 are mostly the ones with a low signal-to-noise ratio (SNR). This is due to the low concentration of the reagent and the use of the spectrometer with a relatively low field (300 MHz) for such kind of investigations. These experimental conditions were chosen on purpose to demonstrate the behavior of the method at its sensitivity limit. Of course, higher fields would solve the high noise problem, but the data at 300 MHz are the real stress test to the method and in our opinion by far more impactful. The low SNR can also be noticed on the integrals shown in Figure 3(d), where the noise influence manifests itself in the form of oscillations that are caused by the so-called intrinsic SNR.^55^

Additionally, it is worth pointing out that the broadening of peaks typical for polymers has still allowed the separation of the peaks in HSQC spectra even at 300 MHz. Of course, in some cases of extreme broadening the peak overlap can cause additional problems in the analysis of the results. However, in our opinion the limits shown here on this example are especially promising for the broad applicability of the method presented.

The assigned integrals from HSQC have been used to calculate the average mass of the reactants, which was used later as reference data for calibration of the mass from the TR-DOSY experiments.

In Figure 4, we have presented the time dependence of the average mass calculated from TR-NUS peak intensities and from TR-DOSY diffusion coefficients. The curves are in substantial agreement. However, during the first 6 h of the reaction, the diffusion coefficient values are disturbed due to heating from the light source, which causes unstable temperature. This effect manifests itself in the deviation from the TR-NUS data. The disturbance is extended in time due to interleaved acquisition, which causes a single frame to be averaged over 4.8 h. After that, the temperature stabilizes. Additionally, the similar curve obtained from the sequential TR-DOSY experiment proves that the assignment of peaks in the HSQC experiment was correct. Both curves show that maximum average mass is achieved at the 28th hour of our reaction. Later, the average mass drops due to precipitation of higher *n*-mers, which are insoluble. Overall, the presented data show that despite low concentrations of samples, broad NMR lines, and a relatively low field of 300 MHz, the combination of TR-NUS and TR-DOSY can be successfully applied. In the case of samples with higher SNR, of course, the assignment could be potentially confirmed using additional methods, such as spatially encoded 3D DOSY^56,57^ or TR adaptation of HSQC-iDOSY.^58,59^

## Conclusion

The synergy between both time-resolved methods allows us to understand the photopolymerization process of H2banthbn. Using only diffusion methods, we would have only a crude idea about the average mass of the system, and obtaining the information about particular *n*-mers would be close to impossible. The HSQC data allow us to track the concentration changes of each *n*-mer, providing us with quite compelling information about the system. That being said, the assignment of the peaks without the confirmation from the diffusion data would be ambiguous. The use of both methods allows us to have certainty about the assignment and, therefore, to better understand the system. Finally, the presented comprehensive methodology was demonstrated on a challenging system in terms of concentration, line widths, and magnetic field. The presented approach is general and can be used for different types of chemical reactions, especially the polymerization reactions and photoreactions.

## Supporting Information Available

Average molecular mass calculated from TR-DOSY, a picture of the reaction mixture in the NMR tube after the reaction, HRMAS results, discussion about pulsed field gradient sampling, schemes of pulse sequences and acquisition script (PDF)
Animation showing the progress of photopolymerization studied with the interleaved acquisition (MP4)

## Notes

All experimental data and analysis scripts are deposited in ZENODO, DOI:10.5281/zenodo.6572008.
