# Supplementary material for: Deeper Insight into Photopolymerization: The Synergy of Time-Resolved Nonuniform Sampling and Diffusion NMR
Source: J Am Chem Soc. 2022 Jul 19;144(30):13938–45. doi: 10.1021/jacs.2c05944 (PMC9354252; doi:10.1021/jacs.2c05944)
Supplement: Supplementary file 1 — ja2c05944_si_001.pdf [file ja2c05944_si_001.pdf]

Supporting Information: Deeper Insight into Photopolymerization: The Synergy of Time-Resolved Non-Uniform Sampling and Diffusion NMR

Kristina Kristinaityte ,[†] Adam Mames,[†] Mariusz Pietrzak,[†] Franz F. Westermair,[‡] Wagner Silva,[‡] Ruth M. Gschwind,[‡] Tomasz Ratajczyk,[†] and Mateusz Urbańczyk[*,†]

†*Institute of Physical Chemistry, Polish Academy of Sciences, Kasprzaka 44/52, 01-224 Warsaw, Poland*

‡*Faculty of Chemistry and Pharmacy, Univeristy of Regensburg, Universitätsstraße 31, 93053 Regensburg, Germany*

E-mail: murbanczyk@ichf.edu.pl

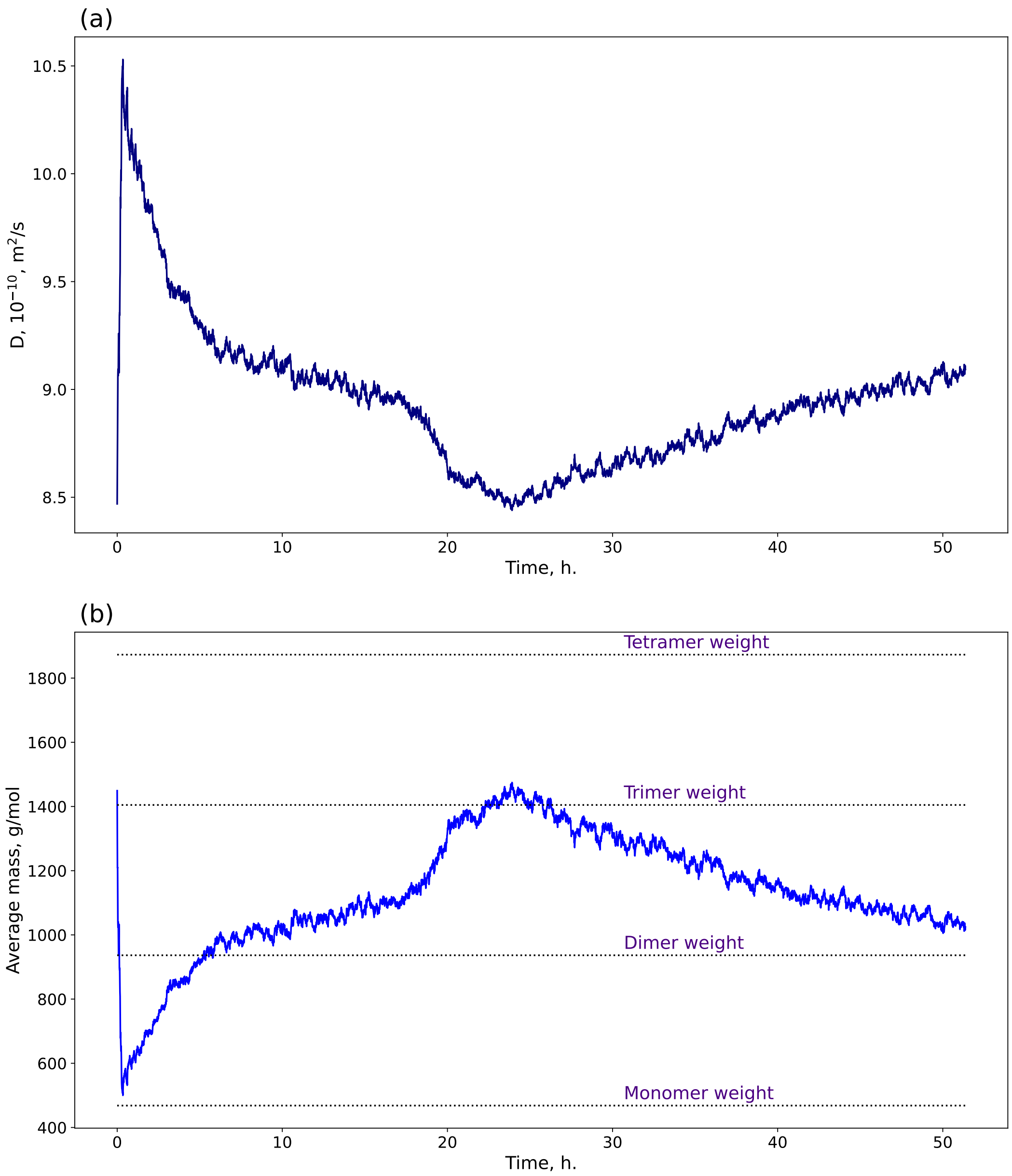

Figure SI.1: a) The diffusion coefficient time dependence calculated from sequential TR-DOSY monitoring of photo-polymerization of H2banthb; b) The average mass time dependence calculated from b). The mass calibration was done using diffusion coefficients and masses of anthracene, dimer of anthracene and H2banthb.

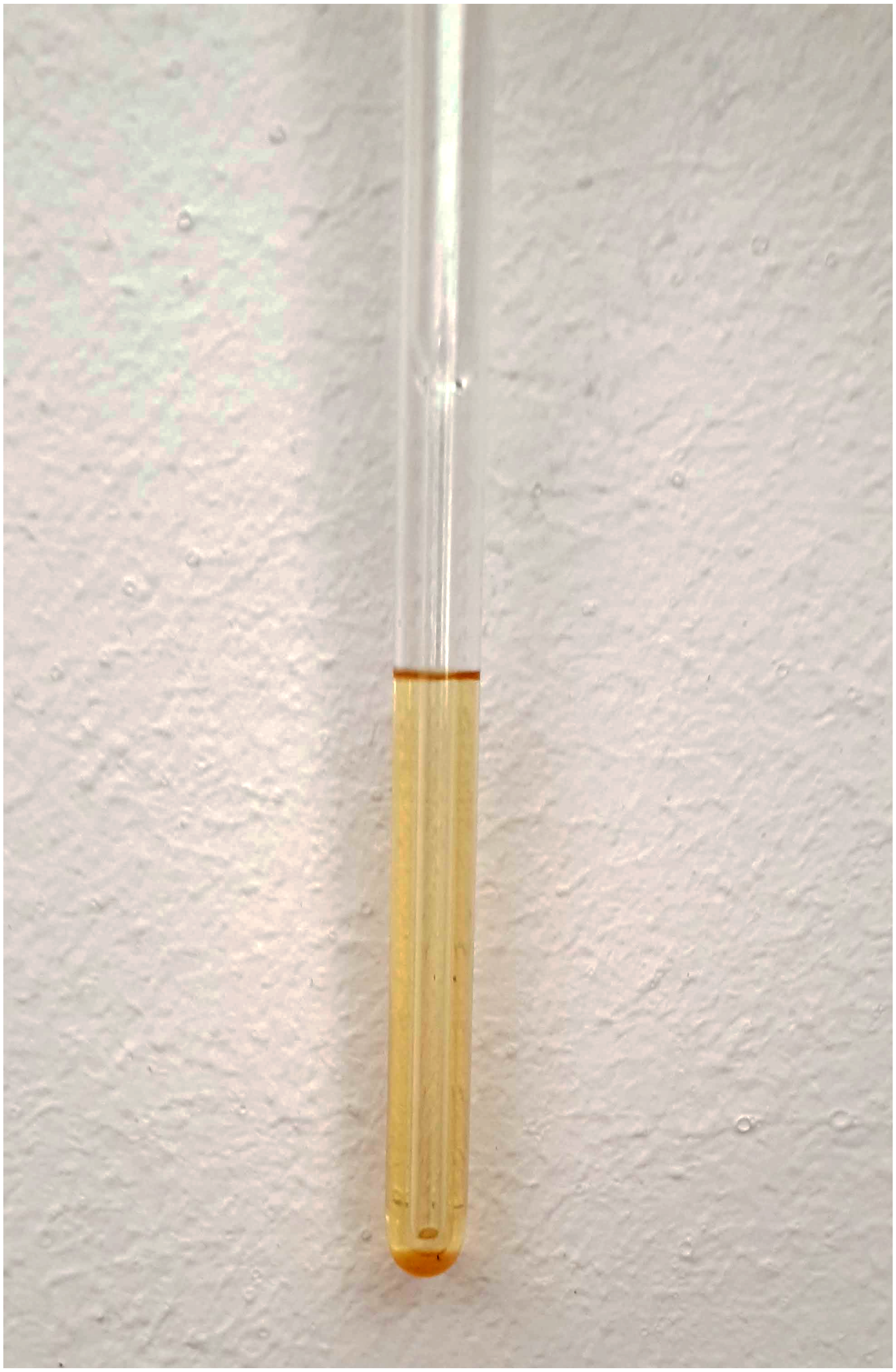

Figure SI.2: Picture of the NMR tube after reaction. On the bottom of the tube there is visible precipitate from the higher mass polymers.

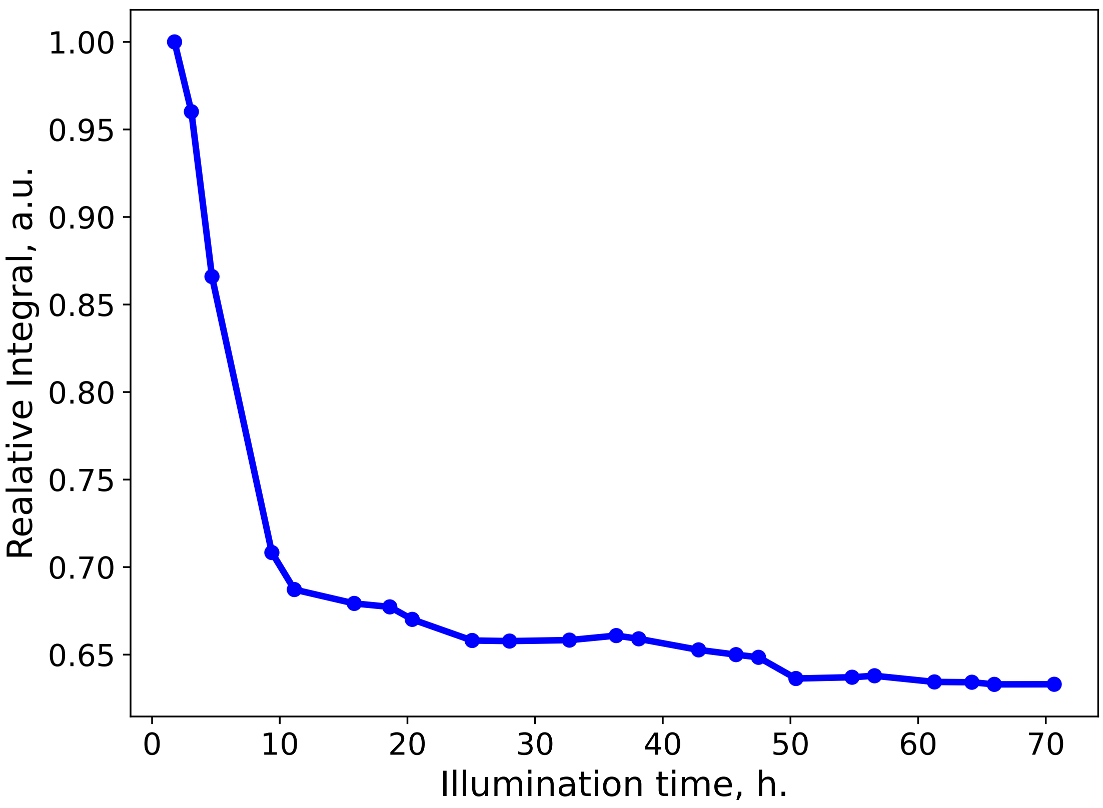

Figure SI.3: The relative integral of the ^{1}H NMR spectrum taken from diffusion data with the lowest gradient strength.

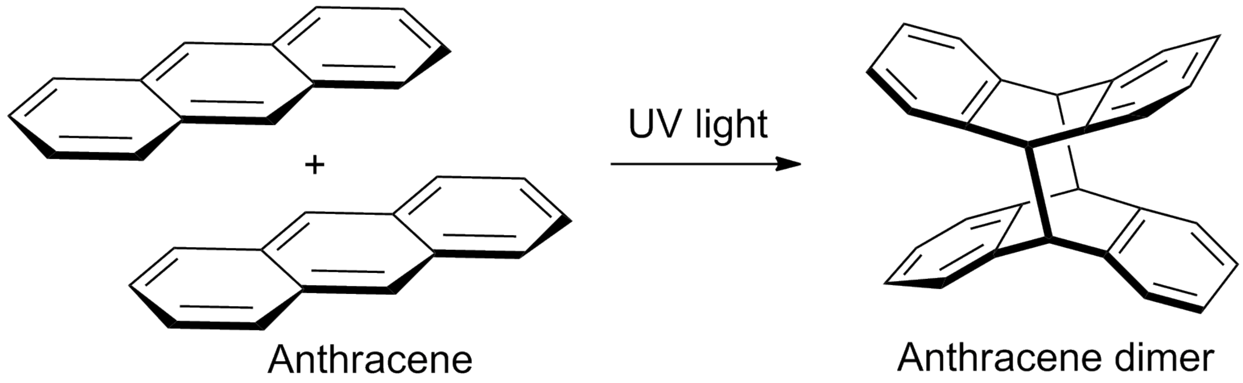

Figure SI.4: The scheme of the reaction of photo-dimerization of anthracene. Used for mass calibration.

HRMAS of H2banthb

Single Mass Analysis
Tolerance = 3.0 mDa / DBE: min = -1.5, max = 300.0
Element prediction: Off
Number of isotope peaks used for i-FIT = 3
Monoisotopic Mass, Even Electron Ions
20 formula(e) evaluated with 1 results within limits (all results (up to 1000) for each mass)

Elements Used:
C: 0-100 H: 0-100 N: 0-2

Mass	Calc. Mass	mDa	PPM	DBE	Formula	i-FIT	i-FIT Norm	Fit Conf %	C	H	N
469.2643	469.2644	-0.1	-0.2	19.5	C34 H33 N2	960.3	n/a	n/a	34	33	2

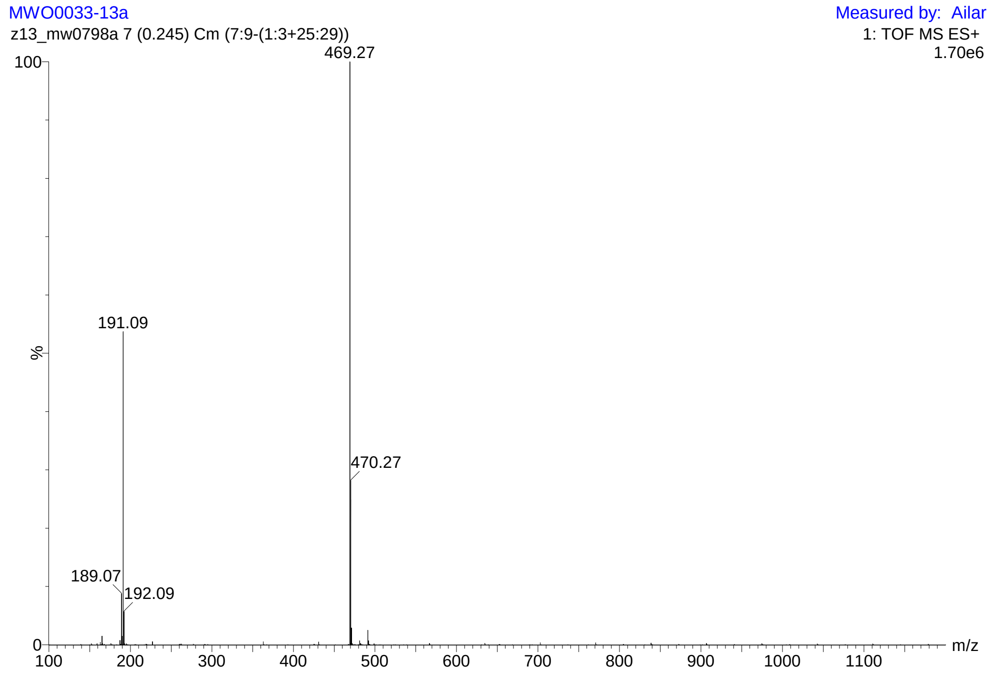

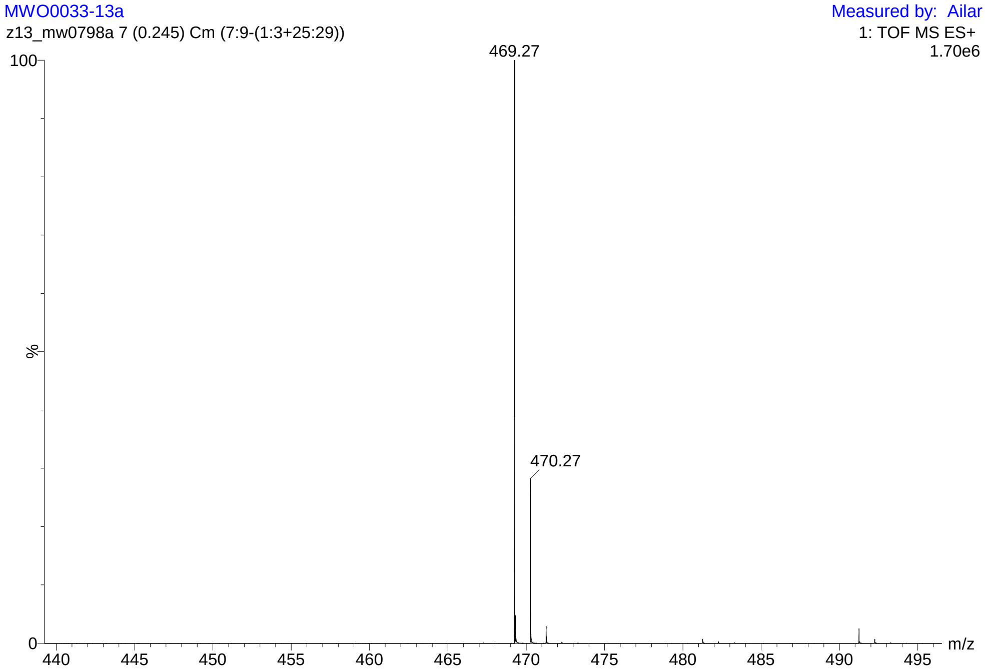
MWO0033-13a
Measured by: Ailar
z13_mw0798a 7 (0.245) Cm (7:9-(1:3+25:29))
1: TOF MS ES+
1.70e6
469.27
470.27
100
%
0
m/z
440
445
450
455
460
465
470
475
480
485
490
495

The choice of gradient sampling

The gradient range was chosen manually based on a series of simulated diffusion decays 7e-10,8e-10,9e-10,8.5e-10,1e-9 m^2/s. The sampling was chosen to be exponential and to guarantee the best separation of decays for aforementioned diffusion coefficients (See Figure SI.5).

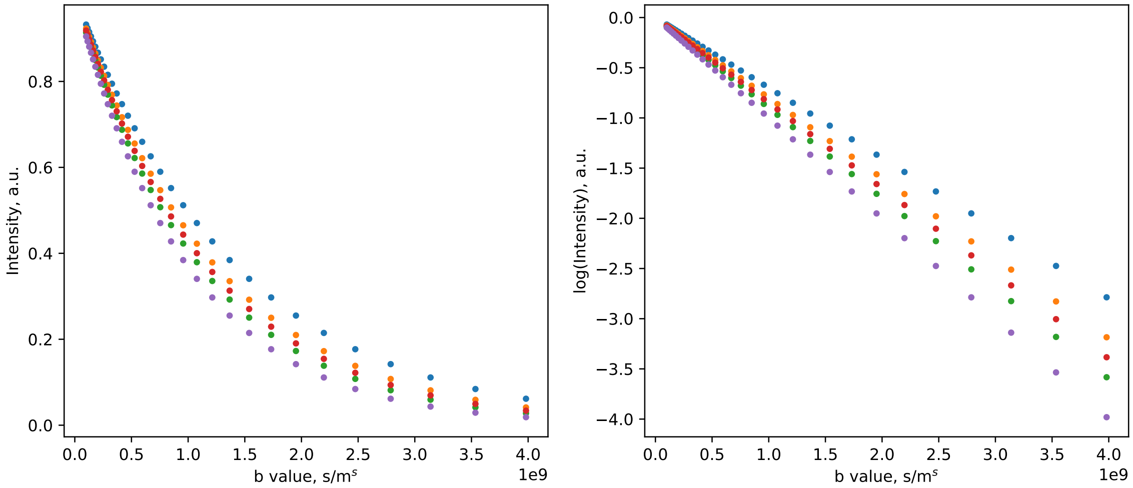

Figure SI.5: The simulated decays of the diffusion coefficients: 7e-10,8e-10,9e-10,8.5e-10,1e-9 m^2/s

Pulse sequences used

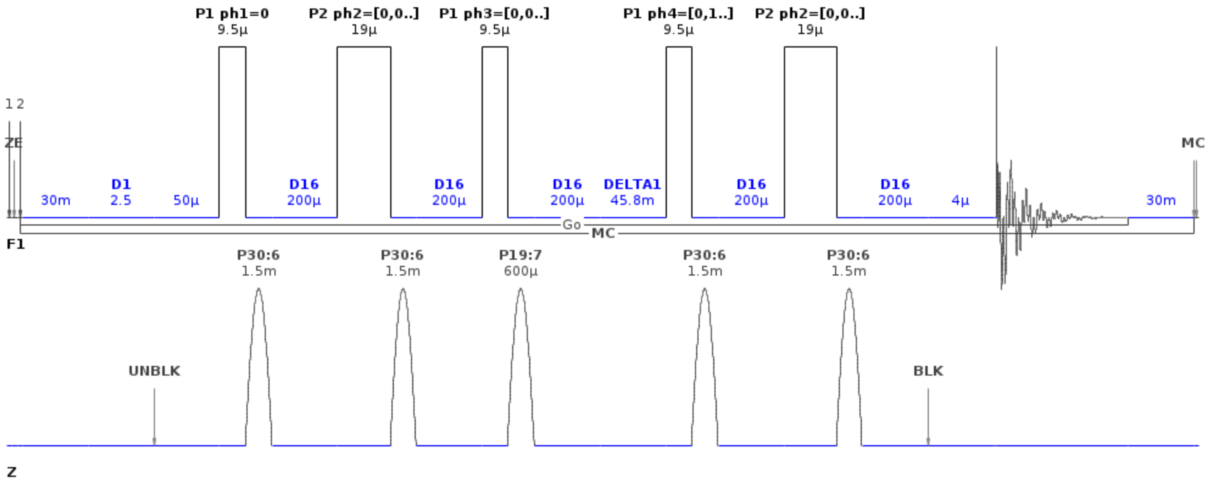

Figure SI.6: Pulse sequence: stebpgp1s1d

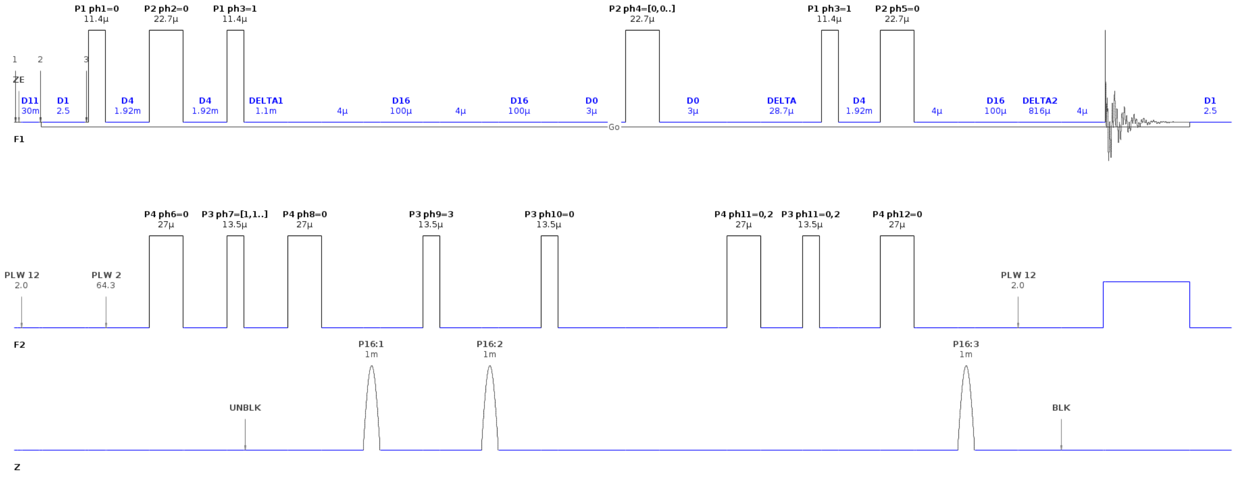

Figure SI.7: Pulse sequence: hsqcgpph

Acquisiton code based on TReNDS:

```
#!/usr/local/bin/jython
# -*- coding: utf-8 -*-
import os
import shutil
import threading
import time
import datetime
from java.awt import *
from javax.swing import *
from javax.swing.filechooser import FileNameExtensionFilter



class TopTReND(JFrame):
```

```
    def __init__(self):
        super(TopTReND, self).__init__()
        self.initUI()

    def initUI(self):
        stepsTXT = JLabel('Steps for IL acquisition:   ')
        Spacer1 = JLabel(' \n')
        Exp1 = JLabel('\n\n\n*******************2D exp No
        ↪  1*******************\n\n\n')
        Exp2 = JLabel('\n*******************2D exp No
        ↪  2*******************\n')
        Exp3 = JLabel('\n*******************2D exp No
        ↪  3*******************\n')
        ExpTR = JLabel('\n*******************TR DOSY exp
        ↪  *******************\n')

        Spacer = JLabel(' \n\n ****************************************\n\n
        ↪  ')
        Spacer2 = JLabel(' \n\n ****************************************\n\n
        ↪  ')
        numof = JLabel('Number of 2D exp:  ')
        self.samp = JButton('Choose sampling schedule',
        ↪  actionPerformed=self.FileChooser)
        self.samp2 = JButton('Choose sampling schedule',
        ↪  actionPerformed=self.FileChooser2)
        self.samp3 = JButton('Choose sampling schedule',
        ↪  actionPerformed=self.FileChooser3)
```

```
        self.Patern2D = JButton('Choose experiment to be used as 2D template', actionPerformed=self.TwodimPatern)
        self.Patern2D_1 = JButton('Choose experiment to be used as 2D template', actionPerformed=self.TwodimPatern2)
        self.Patern2D_2 = JButton('Choose experiment to be used as 2D template', actionPerformed=self.TwodimPatern3)
        self.Patern2D_tr = JButton('Choose experiment to be used as TR DOSY template', actionPerformed=self.TwodimPaternTR)
        self.sampTR = JButton('Choose sampling schedule', actionPerformed=self.FileChooserTR)

        self.Patern1D = JButton('Choose experiment to be used as 1D template', actionPerformed=self.OnedimPatern)
        self.SaveDir = JButton('Choose Directory to save data', actionPerformed=self.DirChooser)
        self.RunBTN = JButton('Start Acquisition!', actionPerformed=self.onRunt)
        self.StopBTN = JButton('Stop Acquisition!', actionPerformed=self.onStop)
        self.StopBTN.setEnabled(False)
        spin2 = SpinnerNumberModel(100.0, 1.0, 999999.0, 1.0)
        spin = SpinnerNumberModel(1.0, 1.0, 3.0, 1.0)
        self.numofexp = JSpinner(spin)
        self.steps = JSpinner(spin2)
        self.twodionly = JCheckBox('2D acquisition only')
        self.setTitle('TReNDS  Acquisition module for TopSpin with TR-DOSY') # create window with title
        self.setSize(600, 700)  # set window size x, y
```

```
        self.panel1 = JPanel()
        self.panel2 = JPanel()
        self.panel4 = JPanel()
        self.panel1.setAlignmentX(Component.LEFT_ALIGNMENT)
        self.panel2.setAlignmentX(Component.LEFT_ALIGNMENT)
        self.panel4.setAlignmentX(Component.LEFT_ALIGNMENT)
        layoutout1 = BoxLayout(self.panel1, BoxLayout.Y_AXIS)
        layoutout2 = BoxLayout(self.panel2, BoxLayout.X_AXIS)
        layoutout3 = BoxLayout(self.panel4, BoxLayout.X_AXIS)
        self.panel1.setLayout(layoutout1)
        self.panel2.setLayout(layoutout2)
        self.panel4.setLayout(layoutout3)
        self.setLayout(FlowLayout())  # layout manager for horizontal alignment
        self.add(self.panel1)
        self.panel1.add(self.panel2)
        self.panel1.add(JLabel('\n'))
        self.panel1.add(self.Patern1D)
        self.panel1.add(self.twodionly)
        self.panel1.add(JLabel('\n'))
        self.panel1.add(Exp1)
        self.panel1.add(JLabel('\n'))
        self.panel1.add(self.samp)
        self.panel1.add(self.Patern2D)
        self.panel1.add(JLabel('\n'))
        self.panel1.add(Exp2)
        self.panel1.add(JLabel('\n'))
        self.panel1.add(self.samp2)
```

```
        self.panel1.add(self.Patern2D_1)
        self.panel1.add(JLabel('\n'))
        self.panel1.add(Exp3)
        self.panel1.add(JLabel('\n'))
        self.panel1.add(self.samp3)
        self.panel1.add(self.Patern2D_2)
        self.panel1.add(JLabel('\n'))
        self.panel1.add(ExpTR)
        self.panel1.add(JLabel('\n'))
        self.panel1.add(self.sampTR)
        self.panel1.add(self.Patern2D_tr)
        self.panel1.add(JLabel('\n'))
        self.panel1.add(Spacer2)
        self.panel1.add(JLabel('\n'))
        self.panel1.add(self.SaveDir)
        self.panel1.add(Spacer1)
        self.panel1.add(Spacer)
        self.panel1.add(self.panel4)
        self.panel4.add(self.RunBTN)
        self.panel4.add(JLabel('\t'))
        self.panel4.add(self.StopBTN)
        self.panel2.add(stepsTXT)
        self.panel2.add(self.steps)
        self.panel2.add(numof)
        self.panel2.add(self.numofexp)
        self.setDefaultCloseOperation(JFrame.DISPOSE_ON_CLOSE)
        self.setVisible(True)
        self.setAlwaysOnTop(True)
```

```
    def FileChooser(self, e):
        self.setAlwaysOnTop(False)

        chooseFile = JFileChooser()
        filter1 = FileNameExtensionFilter("sampling schedule", [".sch"])
        chooseFile.addChoosableFileFilter(filter1)

        ret = chooseFile.showDialog(JPanel(), "Choose file")

        if ret == JFileChooser.APPROVE_OPTION:
            file = chooseFile.getSelectedFile()
            self.sampfile = file.getCanonicalPath()
            mes = "Sampling file: " + self.sampfile + " have been chosen"
            SHOW_STATUS(message=mes)
            self.samp.setText(mes)
        self.setAlwaysOnTop(True)

    def FileChooser2(self, e):
        self.setAlwaysOnTop(False)

        chooseFile = JFileChooser()
        filter1 = FileNameExtensionFilter("sampling schedule", [".sch"])
        chooseFile.addChoosableFileFilter(filter1)

        ret = chooseFile.showDialog(JPanel(), "Choose file")

        if ret == JFileChooser.APPROVE_OPTION:
```

```
            file = chooseFile.getSelectedFile()
            self.sampfile2 = file.getCanonicalPath()
            mes = "Sampling file: " + self.sampfile + " have been chosen"
            SHOW_STATUS(message=mes)
            self.samp2.setText(mes)
        self.setAlwaysOnTop(True)

    def FileChooser3(self, e):
        self.setAlwaysOnTop(False)

        chooseFile = JFileChooser()
        filter1 = FileNameExtensionFilter("sampling schedule", [".sch"])
        chooseFile.addChoosableFileFilter(filter1)

        ret = chooseFile.showDialog(JPanel(), "Choose file")

        if ret == JFileChooser.APPROVE_OPTION:
            file = chooseFile.getSelectedFile()
            self.sampfile3 = file.getCanonicalPath()
            mes = "Sampling file: " + self.sampfile + " have been chosen"
            SHOW_STATUS(message=mes)
            self.samp3.setText(mes)
        self.setAlwaysOnTop(True)

    def FileChooserTR(self, e):
        self.setAlwaysOnTop(False)

        chooseFile = JFileChooser()
```

```
        filter1 = FileNameExtensionFilter("gradient schedule", [".txt"])
        chooseFile.addChoosableFileFilter(filter1)

        ret = chooseFile.showDialog(JPanel(), "Choose file")

        if ret == JFileChooser.APPROVE_OPTION:
            file = chooseFile.getSelectedFile()
            self.sampfileTR = file.getCanonicalPath()
            mes = "Sampling file: " + self.sampfileTR + " have been chosen"
            SHOW_STATUS(message=mes)
            self.sampTR.setText(mes)
        self.setAlwaysOnTop(True)
    def DirChooser(self, e):
        self.setAlwaysOnTop(False)

        chooseFile = JFileChooser()
        chooseFile.setFileSelectionMode(JFileChooser.DIRECTORIES_ONLY)
        filter1 = FileNameExtensionFilter("sampling schedule", [".sch"])
        chooseFile.addChoosableFileFilter(filter1)

        ret = chooseFile.showDialog(JPanel(), "Choose file")

        if ret == JFileChooser.APPROVE_OPTION:
            file = chooseFile.getSelectedFile()

            self.dirsave = file.getCanonicalPath()
            mes = "Acquired data will be saved in : " + self.dirsave + " directory"
```

```
            SHOW_STATUS(message=mes)
            self.SaveDir.setText(mes)
            self.setAlwaysOnTop(True)

            # def TwoDimTemp(e):
            # def TwoDimTemp(e):

    def TwodimPatern(self, e):
        self.setAlwaysOnTop(False)

        self.twodimpath = DATASET_DIALOG(
            "Choose 2D experiment pattern (DO NOT USE FIND OPTION!)")  # PROBLEM WITH FIND BUTTON!!!
        mes = '2D template is: ' + self.twodimpath[3] + '/' + self.twodimpath[0] + '/' + self.twodimpath[1] + '/' + \
              self.twodimpath[2]
        SHOW_STATUS(message=mes)
        # DATASET_DIALOG("Choose 1D experiment pattern", CURDATA())
        self.Patern2D.setText(mes)
        self.setAlwaysOnTop(True)

    def TwodimPatern2(self, e):
        self.setAlwaysOnTop(False)

        self.twodimpath2 = DATASET_DIALOG(
            "Choose 2D experiment pattern (DO NOT USE FIND OPTION!)")  # PROBLEM WITH FIND BUTTON!!!
```

```
        mes = '2D template is: ' + self.twodimpath2[3] + '/' + self.twodimpath2[0] + '/' + self.twodimpath2[1] + '/' + \
              self.twodimpath2[2]
        SHOW_STATUS(message=mes)
        # DATASET_DIALOG("Choose 1D experiment pattern", CURDATA())
        self.Patern2D_1.setText(mes)
        self.setAlwaysOnTop(True)

    def TwodimPaternTR(self, e):
        self.setAlwaysOnTop(False)

        self.twodimpathTR = DATASET_DIALOG(
            "Choose 2D experiment pattern (DO NOT USE FIND OPTION!)")  # PROBLEM WITH FIND BUTTON!!!
        mes = '2D template is: ' + self.twodimpathTR[3] + '/' + self.twodimpathTR[0] + '/' + self.twodimpathTR[1] + '/' + \
              self.twodimpathTR[2]
        SHOW_STATUS(message=mes)
        # DATASET_DIALOG("Choose 1D experiment pattern", CURDATA())
        self.Patern2D_tr.setText(mes)
        self.setAlwaysOnTop(True)
    def TwodimPatern3(self, e):
        self.setAlwaysOnTop(False)

        self.twodimpath3 = DATASET_DIALOG(
            "Choose 2D experiment pattern (DO NOT USE FIND OPTION!)")  # PROBLEM WITH FIND BUTTON!!!
```

```
        mes = '2D template is: ' + self.twodimpath3[3] + '/' + self.twodimpath3[0] + '/' + self.twodimpath3[1] + '/' + \
              self.twodimpath3[2]
        SHOW_STATUS(message=mes)
        # DATASET_DIALOG("Choose 1D experiment pattern", CURDATA())
        self.Patern2D_2.setText(mes)
        self.setAlwaysOnTop(True)

        # self.twodimpath=DATASET_DIALOG("Choose 1D experiment pattern", CURDATA())

    def OnedimPatern(self, e):
        self.setAlwaysOnTop(False)

        self.onedimpath = DATASET_DIALOG("Choose 1D experiment pattern (DO NOT USE FIND OPTION!)")
        mes = '1D template is: ' + self.onedimpath[3] + '/' + self.onedimpath[0] + '/' + self.onedimpath[1] + '/' + \
              self.onedimpath[2]
        SHOW_STATUS(message=mes)
        # DATASET_DIALOG("Choose 1D experiment pattern", CURDATA())
        # self.twodimpath=DATASET_DIALOG("Choose 1D experiment pattern", CURDATA())
        self.Patern1D.setText(mes)
        self.setAlwaysOnTop(True)

    def onStop(self, e):
        self.eventkill.set()
```

```python
        self.StopBTN.setEnabled(False)
        self.RunBTN.setEnabled(True)
        self.RunBTN.setText("Start Acquisition")

    def onRunt(self, e):
        self.StopBTN.setEnabled(True)
        twoditemp = self.twodimpath
        if self.numofexp.getValue() < 3:
            self.sampfile3 = ''
            self.twodimpath3 = ''
        if self.numofexp.getValue() < 2:
            self.sampfile2 = ''
            self.twodimpath2 = ''
        if self.twodionly.isSelected():
            self.onedimpath = ''
        self.eventkill = threading.Event()
        self.first = threading.Thread(target=self.acquisitionloop,
                                      args=(self.steps.getValue(),
                                      ↪   self.sampfile,self.twodimpathTR,
                                      ↪   self.twodimpath, self.onedimpath,
                                            self.dirsave,
                                            ↪   self.twodionly.isSelected(),
                                            ↪   self.sampfile2,
                                            ↪   self.sampfile3,
                                            ↪   self.sampfileTR,
                                            self.twodimpath2,
                                            ↪   self.twodimpath3,
                                            ↪   self.numofexp.getValue(),
```

```
                                                                    self.eventkill))
        # first.setDaemon(True)
        self.first.start()

    def acquisitionloop(self, steps, sampfile, twoditempTR, twoditemp,
    ↪   oneditemp, SaveDir, TwoDiOnly, sampfile2, sampfile3, sampfileTR,
                        twoditemp2, twoditemp3, numofexp, eventkill):
        self.RunBTN.setEnabled(False)
        self.create_interleave_file_structure(SaveDir, numofexp)  # creates
        ↪   subfolders like Acquired/1D Acquired/2D
        # with open(sampfile) as f:
        #     schedule1 = f.readlines()
        f = open(sampfile, 'r')
        schedule1 = f.readlines()
        f.close()
        f = open(sampfileTR, 'r')
        scheduleTR = f.readlines()
        f.close()
        shutil.copy2(sampfileTR, SaveDir + '/Acquired/DOSY/gradients.txt')

        shutil.copy2(sampfile, SaveDir + '/Acquired/2D/sampling.sch')
        if int(numofexp) == 3:
            shutil.copy2(sampfile3, SaveDir + '/Acquired/2D_2/sampling.sch')
            # with open(sampfile3) as f:
            #     schedule3 = f.readlines()
            f = open(sampfile3, 'r')
            schedule3 = f.readlines()
            f.close()
```

```
        if int(numofexp) == 3 or int(numofexp) == 2:
            shutil.copy2(sampfile2, SaveDir + '/Acquired/2D_1/sampling.sch')
            # with open(sampfile2) as f:
            #     schedule2 = f.readlines()
            f = open(sampfile2, 'r')
            schedule2 = f.readlines()
            f.close()
        W = 0
        itr=0
        for i in xrange(2, 2 * int(steps) + 2, 2):


            if not eventkill.wait(1):
                # txtbtn = "Acq. running. Step  " + str(i / 2) + "/" +
                ↪  str(steps)
                # self.RunBTN.setText(txtbtn)
                for k in range(0, int(numofexp)):
                    mess = "Step" + str(i / 2)
                    #SHOW_STATUS(message=str(schedule[i]))

                    time.sleep(5)
                    txtbtn = "Acq. running. Step  " + str(i / 2) + "/" +
                    ↪  str(steps)
                    self.RunBTN.setText(txtbtn)
                    # start 2D
                    if k == 0:
                        schedule = schedule1
```

```
    self.copydataTo(twoditemp, SaveDir + '/Acquired',
    ↪  '2D', i / 2)
    self.openFolderPath(SaveDir + '/Acquired/2D/' + str(i
    ↪  / 2) + '/pdata/' + twoditemp[2])
    time.sleep(5)
    fname = SaveDir + '/Acquired/2D/' + str(i / 2) +
    ↪  '/nusILlist'
elif k == 1:
    schedule = schedule2
    self.copydataTo(twoditemp2, SaveDir + '/Acquired',
    ↪  '2D_1', i / 2)
    self.openFolderPath(SaveDir + '/Acquired/2D_1/' +
    ↪  str(i / 2) + '/pdata/' + twoditemp2[2])
    time.sleep(5)
    fname = SaveDir + '/Acquired/2D_1/' + str(i / 2) +
    ↪  '/nusILlist'
elif k == 2:
    schedule = schedule3
    self.copydataTo(twoditemp3, SaveDir + '/Acquired',
    ↪  '2D_2', i / 2)
    self.openFolderPath(SaveDir + '/Acquired/2D_2/' +
    ↪  str(i / 2) + '/pdata/' + twoditemp3[2])
    time.sleep(5)
    fname = SaveDir + '/Acquired/2D_2/' + str(i / 2) +
    ↪  '/nusILlist'
f = open(fname, 'w+')
f.write(schedule[W])
```

```
f.write(schedule[W + 1])  # retest it when the new generator is ready
f.close()
try:
    topspin_path = sys.getEnviron()['XWINNMRHOME']  # topspin < 3,1
except:
    topspin_path = sys.registry['XWINNMRHOME']  # topspin > 3.1
f = open(topspin_path + '/exp/stan/nmr/lists/vc/trendnls.txt', 'w+')
f.write(schedule[W])
f.write(schedule[W + 1])  # retest it when the new generator is ready
# self.RunBTN.setText(str(schedule[i/2]))
f.close()
self.sendcommand('fntype non-uniform_sampling')
self.sendcommand('NUSpoints 2')
self.sendcommand('DS 0')
self.putpar('NUSLIST', 'trendnls.txt')  # ON test xcmd gives error so putpar is safer
time.sleep(5)
self.sendcommand('zg')
time.sleep(10)
if k == 0:
    self.acqtest(i, '2D',
```

```python
                                SaveDir)  # KEEPS loop with sleep until audita.txt shows that acquisition is running
                elif k == 1:
                    self.acqtest(i, '2D_1',
                                SaveDir)  # KEEPS loop with sleep until audita.txt shows that acquisition is running
                elif k == 2:
                    self.acqtest(i, '2D_2',
                                SaveDir)  # KEEPS loop with sleep until audita.txt shows that acquisition is running

            W += 2
            if not TwoDiOnly:
                self.copydataTo(oneditemp, SaveDir + '/Acquired', '1D', i / 2)
                self.openFolderPath(SaveDir + '/Acquired/1D/' + str(i / 2) + '/pdata/' + oneditemp[2])
                time.sleep(10)
                self.sendcommand('zg')
                time.sleep(10)
                self.acqtest(i, '1D', SaveDir)
            # TR DOSY part
            self.copydataTo(twoditempTR, SaveDir + '/Acquired', 'DOSY', i / 2)
```

```
                self.openFolderPath(SaveDir + '/Acquired/DOSY/' + str(i / 2) + '/pdata/' + twoditemp[2])
                time.sleep(5)

                self.sendcommand('GPZ6 ' +str(scheduleTR[itr][:-3]))
                time.sleep(5)
                itr+=1
                self.sendcommand('zg')
                time.sleep(10)

                self.acqtest(i, 'DOSY', SaveDir)

                # fname = SaveDir + '/Acquired/2D/' + str(i / 2) + '/nusILlist'

        self.RunBTN.setText("Acquisition Complete")
        self.RunBTN.setEnabled(True)

    def acqtest(self, i, Mode, SaveDir):
        AcqActive = True
        while AcqActive:
            if 'acquisition in progress' in open(SaveDir + '/Acquired/' + Mode + '/' + str(
                    i / 2) + '/audita.txt').read():
                SHOW_STATUS('Acquisition in progress')
                time.sleep(10)
            else:
                AcqActive = False
```

```
                SHOW_STATUS('Acquisition step finished. Next step')

    def openFolder(self, folderM):

        com = 'RE(["'
        for l in range(0, 3):
            com += folderM[l] + '","'
        com += folderM[l + 1] + '"],"y")'
        SHOW_STATUS(message=com)
        EXEC_PYSCRIPT(com)

    def openFolderPath(self, folderM):
        com = 'RE_PATH("' + folderM + '","y")'
        EXEC_PYSCRIPT(com)

    def savePath(self, folder):
        com = 'WR_PATH("' + folder + '","y")'
        EXEC_PYSCRIPT(com)

    def sendcommand(self, command):
        com = 'XCMD("' + command + '",1)'
        EXEC_PYSCRIPT(com)

    def putpar(self, command, path):
        com = 'PUTPAR("' + command + '",' + '"' + path + '")'
        EXEC_PYSCRIPT(com)

```

```
    def copydataTo(self, Source, Destination, Mode, counter):  # Mode 2D vs 1D counter is from loop
        Source = Source[3] + '/' + Source[0] + '/' + Source[1] + '/'
        # SHOW_STATUS(message=Source)
        # self.openFolder(self, Source)
        Destination += '/' + Mode + '/' + str(counter)
        # SHOW_STATUS(message=Destination)
        # # time.sleep(5)
        shutil.copytree(Source, Destination)

        # self.savePath(Destination)
        # # time.sleep(5)
        if Mode == '1D':
            try:
                os.remove(Destination + '/fid')
            except:
                pass
        elif Mode == '2D':
            try:
                os.remove(Destination + '/ser')
            except:
                pass
        SHOW_STATUS(message='Acq structure created in: ' + Destination)


    def create_interleave_file_structure(self, Directory, numofexp):
        # Directory=check_if_dir(Directory)
        # Creates the folder structure for saving the data in Directory
```

```
        if not (os.path.isdir(Directory)):
            os.makedirs(Directory)
            os.makedirs(Directory + '/Acquired')

            os.makedirs(Directory + '/Acquired/1D')
            os.makedirs(Directory + '/Acquired/2D')
            os.makedirs(Directory + '/Acquired/DOSY')

        else:

            if not (os.path.isdir(Directory + '/Acquired')):
                os.makedirs(Directory + '/Acquired')
                os.makedirs(Directory + '/Acquired/1D')
                os.makedirs(Directory + '/Acquired/2D')
                os.makedirs(Directory + '/Acquired/DOSY')

            else:
                os.rename(Directory + '/Acquired',
                          Directory +
                          ↪ '/Acquired_back_at{:%Y_%b_%d_%H_%M_%S}'.format(
                              datetime.datetime.now()))
                os.makedirs(Directory + '/Acquired')
            if not (os.path.isdir(Directory + '/Acquired/1D')):
                os.makedirs(Directory + '/1D')
            if not (os.path.isdir(Directory + '/Acquired/2D')):
                os.makedirs(Directory + '/Acquired/2D')
        if int(numofexp) == 2 or int(numofexp) == 3:
            if not (os.path.isdir(Directory + '/Acquired/2D_1')):
```

```
                os.makedirs(Directory + '/Acquired/2D_1')
        if int(numofexp) == 3:
            if not (os.path.isdir(Directory + '/Acquired/2D_2')):
                os.makedirs(Directory + '/Acquired/2D_2')


TopTReND()
```
